# The Dangerous Liaison Between Depression and Cardiovascular Disease: A Narrative Review of Epidemiology, Mechanisms, and Clinical Management

**DOI:** 10.3390/jcm15186967

**Published:** 2026-09-09

**Authors:** Francesco Angeli, Matteo Armillotta, Luca Bergamaschi, Carmine Pizzi

**Affiliations:** 1 Department of Medical and Surgical Sciences—DIMEC, University of Bologna, Via Giuseppe Massarenti 9, 40138 Bologna, Italy; 2 Cardiovascular Division, Morgagni-Pierantoni University Hospital, 47121 Forlì, Italy

**Keywords:** depression, cardiovascular disease, coronary artery disease, heart failure, myocardial infarction, inflammation, autonomic dysfunction, psychocardiology, cardiac rehabilitation, antidepressants

## Abstract

Depression and cardiovascular disease (CVD) are two of the heaviest burdens carried by modern medicine, and a growing body of evidence now suggests that they are linked by a bidirectional, mutually reinforcing relationship rather than by a simple cause-and-effect chain. Patients with major depressive disorder appear to carry an elevated risk of incident coronary artery disease, heart failure, and stroke, while patients with established CVD, particularly those recovering from an acute coronary syndrome, frequently go on to develop clinically significant depressive symptoms that worsen prognosis independently of traditional risk factors. Several shared pathophysiological pathways help explain this dangerous liaison, among them autonomic dysfunction, dysregulation of the hypothalamic–pituitary–adrenal axis, chronic low-grade inflammation, platelet hyperreactivity, endothelial dysfunction, and unhealthy behavioral patterns that tend to accumulate once mood suffers. Despite this growing mechanistic understanding, translating this knowledge into effective treatment has proven far from straightforward: several randomized trials of antidepressants and psychotherapy have improved mood, yet they have not consistently reduced hard cardiovascular endpoints, with a handful of notable exceptions. This review brings together the current evidence on epidemiology, biological mechanisms, prognostic implications, and management strategies at the depression–CVD interface, and closes by outlining priorities for future research and for integrated cardiac–behavioral care.

## 1. Introduction

Cardiovascular diseases (CVDs), and in particular ischemic cardiomyopathy, remain the leading cause of death worldwide, representing a significant burden for healthcare systems [1]. Mental health diseases, with depression foremost among them, are projected to become one of the leading causes of disability-adjusted life years within the next decade [2,3].

For a long time, cardiovascular disease and depression were studied and treated as separate entities, with little attention paid to the relationship between them [4]. However, over the past three decades, an increasingly sophisticated body of epidemiological, biological, and clinical research has converged on a striking conclusion: depression and cardiovascular disease are intimately and reciprocally connected, so much so that some authors now speak of a heart–brain axis governing their co-occurrence [5].

On the one hand, depression seems to act as an independent risk factor for the development of coronary artery disease, heart failure, and stroke, with effect sizes that in some analyses are comparable to those of more traditional risk factors such as smoking or dyslipidemia [6,7]. On the other hand, an acute cardiovascular event, most classically a myocardial infarction but also acute heart failure, commonly precipitates a depressive episode, which in turn tends to worsen adherence to secondary prevention and reduce engagement with cardiac rehabilitation and is independently associated with higher rates of reinfarction and death [6,8,9,10]. This reciprocal, self-perpetuating cycle carries important implications for how cardiologists, psychiatrists, and primary care physicians ought to approach the cardiac patient: depression can no longer be regarded as a mere psychological reaction to illness but should instead be treated as a modifiable cardiovascular risk factor [11]. With this in mind, the present review aims to offer a comprehensive and clinically oriented synthesis of the epidemiology, pathophysiology, prognostic significance, and treatment strategies relevant to the management of a depression–cardiovascular disease relationship, drawing on the most recent evidence in the field.

Given the broad, narrative scope of this review, the relevant literature was identified through structured searches of PubMed/MEDLINE and, where appropriate, Scopus, using combinations of the terms “depression,” “cardiovascular disease,” “coronary artery disease,” “heart failure,” and “stroke” with “mechanisms,” “prognosis,” “screening,” and “treatment.” Searches were not restricted to a fixed start date but emphasized publications from the past two decades, and we prioritized meta-analyses, large cohort studies, randomized controlled trials, and current guideline documents, giving preference to the most recent and clinically relevant evidence whenever multiple sources addressed the same question.

## 2. Epidemiology

Cardiovascular disease accounts for a substantial share of global mortality, with coronary artery disease alone responsible for a large proportion of deaths among adults over the age of thirty-five [12,13]. Mental health disorders, depression foremost among them, contribute to millions of deaths annually, largely through their downstream physical health consequences [2,9]. Population-based studies consistently show that depression and cardiovascular disease co-occur far more often than was appreciated in past decades (Table 1). Large, longitudinal, nationwide cohorts have confirmed this overlapping burden: prevalence estimates for clinically significant depressive symptoms among patients with established coronary artery disease or following myocardial infarction range from fifteen to thirty percent. Comparably elevated rates have been observed among patients with chronic heart failure and among stroke survivors, populations in which depressive symptoms frequently go unrecognized, precisely because they overlap with somatic complaints such as fatigue, sleep disturbance, and reduced exercise tolerance [2,9,14].

The relationship, however, is not unidirectional. A growing body of prospective evidence indicates that depression itself independently increases the risk of developing cardiovascular disease in individuals who are initially free of it. Meta-analyses of prospective cohort studies estimate that depression confers roughly a 1.3- to 1.5-fold increased risk of incident coronary heart disease and myocardial infarction, an association that persists after adjustment for traditional cardiovascular risk factors, based on pooled analyses of nearly 900,000 participants across studies with follow-up ranging from two to thirty-seven years [6,15]. Depression has similarly been linked to an increased risk of cardiovascular mortality, with some analyses reporting particularly pronounced associations with fatal heart failure. This bidirectionality—depression as both a consequence of cardiovascular disease and an independent risk factor for its onset—is central to understanding why the two conditions cluster so consistently across populations, and why each may plausibly worsen the trajectory of the other.

Sex differences add a further layer of complexity to this picture. Several studies suggest that women with established cardiovascular disease are particularly vulnerable to developing depression, a finding that may relate to differences in symptom presentation, social support, hormonal factors, and the well-documented tendency for ischemic heart disease to be under-recognized and undertreated in women [9,16]. Age, lifestyle factors, socioeconomic background, comorbid conditions, and genetic predisposition all appear to influence both the strength of the depression–CVD association and its prognostic consequences, a useful reminder that this is not a uniform phenomenon, but one shaped, quite substantially, by individual and social context [9,17,18,19,20].

**Table 1:** Approximate prevalence of clinically significant depressive symptoms across cardiovascular conditions.

Clinical Population	Approx. Prevalence	Typical Onset Window	Main Contributing Factors	Commonly Used Screening Tool	Associated Prognostic Impact
General population (no known CVD)	6–8%	Variable, not disease-related	Baseline psychiatric and psychosocial risk	PHQ-9 in primary care	Reference category
Stable coronary artery disease	15–20%	Insidious, over months to years	Chronic disease burden, lifestyle limitations	PHQ-9 or Beck Depression Inventory	Increase in cardiac events (OR 2.24, 95% CI 1.37–3.60 for 2-year all-cause mortality) [21]
Acute coronary syndrome/post-MI	20–30%	First weeks to months after event	Acute stress response, inflammatory activation, prognostic uncertainty	PHQ-9 during index admission or early follow-up	Independent predictor of reinfarction and mortality (HR 2.4, 95% CI 1.2–4.7 for all-cause mortality) [22]
Chronic heart failure	25–40%	Progressive, tracks with functional decline	Functional limitation, symptom overlap, recurrent hospitalizations	PHQ-9, interpretation limited by somatic overlap	Higher readmission rate and mortality (HR 1.75, 95% CI 1.33–2.30 for all-cause mortality) [23]
Post-stroke	30–35%	Within first year, often 3–6 months	Lesion location, disability, cognitive impairment, social isolation	PHQ-9 or stroke-specific depression scales	Poorer functional recovery, increased mortality (HR 1.59, 95% CI 1.30–1.96 for all-cause mortality) [24]
Post cardiac surgery/heart transplantation	20–35%	Perioperative period and first post-transplant year	Perioperative stress, immunosuppression burden, uncertainty about graft outcome	PHQ-9 with consultation–liaison psychiatry input	Reduced treatment adherence, relevant to graft survival (HR 2.52, 95% CI 1.35–4.71 for 1-year mortality post-transplant) [25]

Abbreviations: CI: confidence interval; CVD: cardiovascular disease; HR: hazard ratio; MI: myocardial infarction; OR: odds ratio; PHQ-9: Patient Health Questionnaire.

## 3. Pathophysiological Mechanisms Linking Depression and Cardiovascular Disease

The biological background of the depression–CVD interplay rests on several interconnected pathophysiological pathways, in which behavioral, autonomic, neuroendocrine, inflammatory, and hemostatic abnormalities converge to increase cardiovascular risk in depressed individuals, while cardiovascular injury itself triggers neurobiological changes that predispose to depression.

### 3.1. Autonomic Nervous System Dysfunction

Depression is characterized by relative sympathetic overactivity and reduced parasympathetic, or vagal, tone, most commonly measured as reduced heart rate variability. This autonomic imbalance lowers the threshold for ventricular arrhythmias, impairs the heart’s ability to adapt to physiological stress, and has been independently associated with increased cardiac mortality in both depressed and non-depressed populations [26,27].

Acute myocardial injury, for its part, can produce lasting autonomic dysregulation through direct damage to cardiac afferent and efferent pathways and through the psychological stress response to the event itself, thereby creating a mechanistic loop between cardiac and mood disturbance [28,29,30,31].

### 3.2. Hypothalamic–Pituitary–Adrenal Axis Dysregulation

Chronic activation of the hypothalamic–pituitary–adrenal (HPA) axis and the associated hypercortisolemia is a well-established feature of major depression and is increasingly recognized as a contributor to adverse cardiovascular remodeling. Sustained cortisol elevation promotes visceral adiposity, insulin resistance, hypertension, and endothelial dysfunction, and it also interacts with the sympathetic nervous system to amplify autonomic imbalance [27,32]. Following an acute coronary event, the physiological stress response itself produces a marked, and sometimes prolonged, activation of the HPA axis, which may help explain why the weeks and months after myocardial infarction represent a period of particular vulnerability to new-onset depression [31,33,34].

### 3.3. Inflammation

Inflammation is likely the most extensively studied mechanistic bridge between depression and cardiovascular disease [8,35]. Myocardial injury triggers the release of damage-associated molecular patterns, which activate macrophages and other immune cells to release pro-inflammatory cytokines including interleukin-6, interleukin-1β, and tumor necrosis factor-α [35]. These same cytokines are elevated in patients with major depressive disorder independent of cardiac status, and are thought to promote depressive symptoms through at least three converging pathways: altered synthesis and metabolism of monoamine neurotransmitters, increased production of kynurenine pathway metabolites that act as agonists at NMDA glutamate receptors, and reduced availability of brain-derived neurotrophic factor [36]. Conversely, chronic inflammation associated with depression accelerates atherosclerotic plaque formation and destabilization, which effectively closes a bidirectional inflammatory loop between mood and vascular disease [4,7,37].

### 3.4. Platelet Activation and Coagulation Abnormalities

Depressed patients, particularly those with concomitant cardiovascular disease, tend to exhibit a pro-thrombotic phenotype characterized by platelet hyperreactivity [38]. Platelets from depressed individuals display increased exposure of anionic phospholipids, enhanced activation of glycoprotein IIb/IIIa receptors, greater granule secretion, and altered expression of serotonin receptor subtypes on the platelet surface [39]. Since platelets store and release serotonin, and since depression is associated with dysregulated serotonergic signaling more broadly, this pathway offers a fairly direct molecular link between mood disorder and the risk of acute thrombotic events [39,40]. Elevated levels of plasminogen activator inhibitor-1 and reduced tissue plasminogen activator activity further tilt the hemostatic balance toward thrombosis in depressed patients, which plausibly contributes to their excess risk of reinfarction and stroke [41].

### 3.5. Endothelial Dysfunction

Endothelial dysfunction, an early and reversible step in atherogenesis, is consistently reported in patients with major depression and is thought to result from the combined effects of inflammation, oxidative stress, and the autonomic dysregulation described above [41,42,43]. Beyond these biological mechanisms, behavioral factors provide an equally important, and arguably more directly modifiable, pathway linking the two conditions. Depression is strongly associated with reduced physical activity, poorer dietary habits, higher rates of smoking and alcohol use, social isolation, and markedly reduced adherence to prescribed cardiovascular medications and rehabilitation programs [44,45]. In patients recovering from myocardial infarction or cardiac surgery, this constellation of behaviors can substantially undermine secondary prevention efforts, quite independently of any direct biological effect of depression on vascular tissue.

### 3.6. Genetic and Pharmacological Considerations

Twin and family studies suggest a partly shared genetic architecture between depression and cardiovascular disease, with variants in genes regulating serotonergic signaling, such as the serotonin transporter gene, implicated in both conditions [44,46].

It is also worth noting that some cardiovascular medications can themselves precipitate or worsen depressive symptoms; beta-blockers, in particular, have historically been associated with depressive side effects, although more recent evidence has softened this association for newer agents, and statins and antiplatelet drugs have also been examined as potential contributors to mood disturbance in susceptible individuals [47,48]. Clinicians should therefore be aware of this possibility whenever a patient’s mood deteriorates after starting a new cardiovascular medication, rather than prematurely attributing all post-cardiac depressive symptoms exclusively to psychological or inflammatory changes.

## 4. Depression as a Cardiovascular Risk Factor

A substantial body of prospective studies suggests that depression, even in the absence of pre-existing heart disease, predicts the future development of coronary artery disease, heart failure, and cerebrovascular events [11,41,45]. Meta-analyses of population-based cohorts have concordantly reported that individuals with clinically significant depressive symptoms carry a relative risk of incident cardiovascular events roughly one and a half times that of non-depressed individuals, an effect that persists after adjustment for traditional risk factors including smoking, physical inactivity, obesity, and diabetes, which suggests that depression contributes to cardiovascular risk through mechanisms only partly explained by behavioral confounding [2,9,16,49].

The dose–response relationship observed in several studies, whereby more severe, chronic, or recurrent depressive episodes confer greater cardiovascular risk than single, mild episodes, lends further support to a causal interpretation rather than to mere reverse causation or confounding by shared risk factors. Large nationwide longitudinal cohorts using cross-lagged panel modeling have managed to formally quantify the relative strength of the depression-to-CVD pathway compared with the CVD-to-depression pathway; some studies suggest that the path from cardiovascular disease to subsequent depression may be numerically stronger, while others find the two directions to be of comparable magnitude, so this remains an area of active investigation, and the balance of directional strength likely varies according to age, population, and the specific cardiovascular endpoint under study [8,50,51].

From a public health perspective, recognizing depression as a modifiable risk factor, analogous to hypertension or dyslipidemia, has important implications for primary prevention. Systematic screening for depressive symptoms in individuals at elevated cardiovascular risk, together with the integration of mental health assessment into routine cardiovascular risk stratification tools, has been proposed as a strategy to close this gap, although its impact on hard outcomes at a population level still remains to be definitively established [17,52,53].

## 5. Cardiovascular Disease as a Trigger for Depression

Conversely, the reverse pathway, that is, cardiovascular disease precipitating depression, is firmly established and recognized in clinical experience, and it has been the subject of the majority of intervention trials conducted in this field so far.

### 5.1. Myocardial Infarction

Depression following acute myocardial infarction is common, affecting a substantial proportion of patients, and it typically emerges within the first weeks to months after the index event [54,55,56,57]. The pathophysiological mechanisms discussed above, namely acute inflammatory activation, HPA axis and autonomic disruption, and the psychological impact of a life-threatening event, all converge during this particularly vulnerable period [27,56,58]. Importantly, post-MI depression is not simply an understandable emotional reaction to a frightening diagnosis; it behaves, prognostically, as an independent risk factor for subsequent cardiac events and mortality, with effect sizes in several studies rivaling those of left ventricular ejection fraction or the extent of coronary disease [54,59].

### 5.2. Heart Failure

Depression is highly prevalent among patients with chronic heart failure, with estimates generally higher than those seen in coronary artery disease alone, likely reflecting the chronic, relapsing, and functionally limiting nature of the syndrome [60,61,62]. In heart failure, depressive symptoms are associated with reduced adherence to fluid and dietary restriction, lower engagement with exercise-based rehabilitation, more frequent hospital readmissions, and increased mortality [60,63]. The overlap between the somatic symptoms of heart failure, such as fatigue, anorexia, sleep disturbance, and reduced exercise capacity, and the somatic criteria for major depressive disorder complicates diagnosis considerably, and likely contributes to under-recognition in routine clinical practice [64,65,66].

### 5.3. Stroke

Post-stroke depression is one of the most extensively studied neuropsychiatric complications of cerebrovascular disease, with a considerable proportion of stroke survivors developing clinically significant depressive symptoms within the first year [41,67,68].

Lesion location, degree of functional disability, cognitive impairment, and social isolation all contribute to the risk of post-stroke depression, which in turn is associated with poorer functional recovery, reduced quality of life, and increased mortality, thereby reinforcing the same bidirectional pattern already observed after myocardial infarction [69].

## 6. Clinical Impact and Prognosis

Regardless of the direction of causality, the presence of comorbid depression and cardiovascular disease is consistently associated with worse clinical outcomes than either condition alone [4,49]. Depressed cardiac patients experience higher rates of major adverse cardiac events, more frequent hospital readmissions, greater healthcare utilization and cost, and increased all-cause and cardiovascular mortality [70]. These associations persist across diverse populations and cardiovascular phenotypes, including coronary artery disease, heart failure, arrhythmias and post-cardiac surgery states, and they appear to be graded according to the severity and persistence of depressive symptoms [63,65,69,71]. Beyond mortality and hard cardiovascular endpoints, comorbid depression substantially degrades quality of life, functional status, and the patient’s ability to participate meaningfully in secondary prevention programs such as cardiac rehabilitation, smoking cessation, and dietary modification. This has direct relevance for clinicians managing complex cardiac patients, including those undergoing cardiac surgery or being evaluated for advanced therapies such as transplantation, in whom psychological resilience and adherence are recognized determinants of long-term success [72,73]. Taken together, the cumulative evidence supports viewing depression not as an incidental comorbidity to be addressed only after cardiovascular issues are stabilized, but rather as a core component of overall cardiovascular risk that warrants proactive identification and management.

## 7. Screening and Diagnosis in Cardiology Practice

Despite strong evidence for the prognostic importance of depression in cardiac patients, systematic screening remains inconsistently implemented in routine cardiology practice. Several major cardiology societies have, at various points, recommended screening for depressive symptoms in patients with coronary artery disease, typically using brief, validated instruments such as the Patient Health Questionnaire-9 (PHQ-9) or the Beck Depression Inventory, both of which can be administered quickly in outpatient or inpatient settings without requiring specialized psychiatric training [51,72].

A recurring challenge in this population is the overlap between the somatic symptoms of depression, such as fatigue, sleep disturbance, appetite change, and psychomotor slowing, and the physical manifestations of cardiovascular disease itself, particularly heart failure [4,65,69,74]. This overlap can lead to both underdiagnosis, when somatic complaints are attributed entirely to cardiac disease, and overdiagnosis, when transient adjustment reactions to a frightening diagnosis are mislabeled as major depressive disorder [75]. Clinical judgment—ideally supported by validated screening tools and, where appropriate, by referral to mental health specialists or consultation–liaison psychiatry services—remains essential to accurate diagnosis. Given that untreated depression carries measurable prognostic weight, a pragmatic approach favoring proactive screening, particularly in the weeks following an acute coronary event or a new heart failure diagnosis, seems justified even where formal guideline recommendations vary in strength.

## 8. Therapeutic Approaches

The translation of mechanistic and epidemiological insight into effective treatment has proven more difficult than initially hoped. A series of landmark randomized controlled trials over the past two decades have tested pharmacological and behavioral interventions for depression in cardiac patients, generally succeeding in improving depressive symptoms but producing mixed results on hard cardiovascular endpoints (Table 2) [76,77,78].

### 8.1. Pharmacological Treatment

The Sertraline Antidepressant Heart Attack (SADHART) trial was among the first to establish the safety of selective serotonin reuptake inhibitors (SSRIs) in patients with recent acute coronary syndrome, demonstrating that sertraline reduced depressive symptoms compared with placebo, particularly among patients with more severe or recurrent depression, without evidence of cardiac harm [79]. Subsequently, the Enhancing Recovery in Coronary Heart Disease (ENRICHD) trial tested a combination of cognitive behavioral therapy and, where needed, antidepressant medication in post-MI patients with depression or low perceived social support; while the intervention produced meaningful improvements in depressive symptoms, it did not reduce the composite endpoint of death or reinfarction [80]. Along similar lines, the MIND-IT trial [81] and the Montreal Heart Attack Readjustment Trial also failed to demonstrate improved survival with their respective psychosocial and pharmacological interventions [82].

More encouraging signals have since emerged, though. The Canadian Cardiac Randomized Evaluation of Antidepressant and Psychotherapy Efficacy (CREATE) trial found that citalopram, but not interpersonal psychotherapy, produced superior improvement in depressive symptoms compared with clinical management alone in patients with coronary artery disease [83]. Particularly notable is the Korean EsDEPACS [84] and DECARD [85] line of research, in which patients with depression following acute coronary syndrome who were treated with escitalopram for six months showed, over a follow-up period extending beyond eight years, a significantly lower rate of major adverse cardiac events compared with placebo, including a marked reduction in recurrent myocardial infarction. This finding represents one of the more compelling pieces of evidence so far that treating depression can plausibly translate into a hard cardiovascular benefit, particularly when treatment is started early and sustained over time.

Less favorable results have been reported in the heart failure population specifically: both the SADHART-CHF [86] trial, which tested sertraline, and the larger MOOD-HF trial [87], which tested escitalopram, failed to demonstrate improvement in cardiovascular outcomes or, in the case of MOOD-HF, even in depressive symptoms beyond what was achieved with placebo and standardized clinical management. This suggests that the pathophysiology and the optimal treatment approach for depression in heart failure may differ meaningfully from that in coronary artery disease. Overall, the SSRI class, and sertraline and escitalopram in particular, is generally regarded as the safest and best-studied pharmacological option for depression in cardiac patients, given the extensive safety data accumulated across these trials and the absence of clinically significant interactions with most cardiovascular medications [84]. Tricyclic antidepressants, by contrast, are generally avoided in this population because of their proarrhythmic and anticholinergic properties [88].

### 8.2. Psychotherapy and Collaborative Care Models

Cognitive behavioral therapy has demonstrated consistent efficacy in reducing depressive symptoms among cardiac patients, as shown in the ENRICHD trial and in subsequent studies, even where these interventions have not translated into reduced hard cardiac endpoints [80].

Collaborative care models, in which a care manager coordinates between primary care, cardiology, and mental health specialists to systematically screen for and treat depression, have shown promise in improving both depressive symptoms and, in some analyses, cardiovascular risk factor control, likely through improved engagement with secondary prevention behaviors rather than through any direct biological effect of psychotherapy on vascular pathology [81].

More recently, the I-CARE randomized clinical trial, conducted across sixteen rural county hospitals in China, showed that an integrative care model combining structured depression management with usual acute coronary syndrome care improved depressive symptoms and other health outcomes compared with usual care alone, supporting the feasibility and value of collaborative care approaches even in low-resource settings [89].

### 8.3. Exercise-Based Cardiac Rehabilitation

Structured exercise, whether performed as a stand-alone intervention or as part of formal cardiac rehabilitation, has an emerging and genuinely favorable evidence base for the treatment of post-cardiac depression. Exercise training improves cardiorespiratory fitness, favorably modulates inflammatory markers and autonomic tone, and produces antidepressant effects of a magnitude comparable to pharmacotherapy in several trials, including analyses derived from the ENRICHD cohort that examined the interaction between exercise participation, depression improvement, and mortality after myocardial infarction [45,90].

Given its additional benefits for traditional cardiovascular risk factors and its excellent safety profile, exercise-based rehabilitation arguably represents the most broadly applicable and cost-effective intervention at the depression–cardiovascular disease interface, and its systematic promotion among depressed cardiac patients should be considered a priority in routine practice [52].

**Table 2:** Selected randomized controlled trials of depression treatment in cardiac and cerebrovascular populations.

Trial	Population	Intervention	Approx. Sample Size	Follow-Up	Main Result
SADHART [79]	Recent ACS with depression	Sertraline vs. placebo	~370	16 weeks	Improved mood, particularly in severe/recurrent depression; no cardiac harm signal (CGI-I responder rate 67% vs. 53%, *p* = 0.01)
ENRICHD [80]	Post-MI with depression or low social support	CBT plus antidepressant when needed	~2480	29 months	Improved depressive symptoms; no reduction in death or reinfarction (event-free survival HR 1.01, 95% CI 0.86–1.18)
MIND-IT [81]	Post-MI with depression	Antidepressant treatment within structured care algorithm	~330	18 months	Modest mood benefit; no demonstrable survival advantage (no significant HAM-D difference vs. care-as-usual on long-term follow-up)
Montreal Heart Attack Readjustment Trial [82]	Post-MI, unselected for baseline depression	Nurse-led psychosocial intervention	~1376	12 months	No overall mortality benefit; possible harm signal in a subgroup of women (all-cause mortality OR 1.41, 95% CI 0.85–2.34 overall; women 10.3% vs. 5.4%, *p* = 0.051)
CREATE [83]	CAD with depression	Citalopram and/or interpersonal psychotherapy	~280	12 weeks	Citalopram superior to placebo for mood; psychotherapy not superior to clinical management (HAM-D difference 3.3 points, 96.7% CI 0.80–5.85, *p* = 0.005)
EsDEPACS/DECARD extension [84,85]	ACS with depression	Escitalopram for six months, long-term follow-up	~300	over 8 years	Lower rate of MACE and reinfarction over long-term follow-up (MACE HR 0.69, 95% CI 0.49–0.96, *p* = 0.03)
SADHART-CHF [86]	Chronic HF with depression	Sertraline vs. placebo	~470	12 weeks	No improvement in cardiovascular outcomes (HDRS score change −7.1 vs. −6.8 points, *p* = 0.89 between groups; composite CV status *p* = 0.78)
MOOD-HF [87]	Chronic HF with depression	Escitalopram vs. placebo	~370	24 months	No improvement in depressive symptoms or cardiovascular outcomes beyond standard care (primary composite endpoint: 116 vs. 119 events; trial stopped early for futility)
Baradaran et al. [91]	Depression before CABG	Escitalopram vs. placebo, perioperative course	~40	2 months	Improved mood and quality of life after surgery (quality-of-life score, *p* = 0.004; depression score, *p* < 0.001)
Yan and Hu [92]	Post-stroke depression	Escitalopram vs. sertraline	~120	12 weeks	Both agents effective and similarly well tolerated (significant HAMD-24 reduction with both agents, *p <* 0.01 vs. baseline; no significant between-group difference)

Abbreviations: CABG: coronary artery bypass graft; CAD: coronary artery disease; CI: confidence interval; CGI-I: Clinical Global Impression Improvement scale; HF: heart failure; HR: hazard ratio; MACE: major adverse cardiac event; OR: odds ratio.

## 9. Special Populations

The depression–cardiovascular disease interplay is not experienced uniformly across the population. Sex, gender, age, and the specific clinical context in which cardiac disease occurs all shape both the risk of developing the comorbid condition and the likelihood of it being recognized and adequately treated.

### 9.1. Gender Differences

Gender differences in the depression–cardiovascular disease relationship are among the most consistently reported, and arguably the most clinically consequential, of the special population effects described in the literature. Depression itself is roughly twice as prevalent in women as in men across the general population, and this disparity persists, and in some studies even widens, among individuals with established cardiovascular disease [9,17]. Women who experience an acute coronary syndrome are more likely than men to develop clinically significant depressive symptoms in the following months, and several cohort studies suggest that depression exerts a proportionally larger effect on cardiac prognosis in women than in men, even after adjustment for baseline disease severity [28,84].

Part of this disparity is likely rooted in the broader, well-documented pattern of underdiagnosis and undertreatment of ischemic heart disease in women. Women more often present with sex-related differences in symptom presentation, are less likely to be referred promptly for invasive evaluation, and remain underrepresented in the pivotal trials that have shaped contemporary cardiovascular guidelines; when a psychiatric comorbidity such as depression is layered onto this pattern, the risk of diagnostic delay and undertreatment compounds further, since fatigue, dyspnea, and chest discomfort attributable to unrecognized coronary disease may instead be misattributed to a mood disorder, or vice versa [87]. Sex-specific ischemic phenotypes that disproportionately affect women, including myocardial infarction with non-obstructive coronary arteries, ischemia with non-obstructive coronary arteries, and stress-induced (Takotsubo) cardiomyopathy, further complicate this picture, since psychological stress and depression appear to play a particularly prominent triggering or aggravating role in these conditions [93,94,95].

Biological and social factors both plausibly contribute to women’s heightened vulnerability. Fluctuations in estrogen and progesterone across the menstrual cycle, the menopausal transition, and pregnancy have been linked to measurable changes in cardiovascular risk, and declining estrogen levels after menopause coincide with a period of increased incidence of both depression and coronary events, suggesting a hormonal contribution to this shared vulnerability window [96,97]. Female-predominant conditions such as autoimmune disease and migraine, which also cluster with depression, may further contribute to this excess risk through shared inflammatory pathways. At the same time, gender-specific psychosocial stressors, including disproportionate caregiving responsibilities, higher rates of partner violence and post-traumatic stress disorder, income inequality, and social role strain, are recognized contributors to cardiovascular risk in women that operate substantially through their effect on mental health [28,98].

Taken together, these observations argue for a sex-sensitive approach to both cardiovascular and psychiatric care: cardiovascular risk assessment in women should explicitly incorporate depression and other female-predominant psychosocial risk factors alongside traditional risk scores, clinicians should keep a low threshold for considering unrecognized ischemic heart disease in women presenting with mood symptoms and cardiac-sounding complaints, and future antidepressant and psychotherapy trials in cardiac populations should be adequately powered, and ideally pre-specified, to detect sex-based differences in treatment response rather than treating sex as an incidental covariate [17].

### 9.2. Older Adults

Older adults with cardiovascular disease face additional challenges, including greater polypharmacy, higher rates of cognitive impairment that can complicate the recognition of depressive symptoms, and generally a more modest response to both pharmacological and psychotherapeutic interventions in the available trial literature, as illustrated by the more equivocal results seen in elderly heart failure populations [99]. Frailty, sensory impairment, and social isolation, all more prevalent in older cardiac patients, further complicate both the detection and the management of comorbid depression, and screening instruments validated in younger populations may perform less reliably in this group [100].

### 9.3. Cardiac Surgery and Transplant Populations

Patients undergoing cardiac surgery, including coronary artery bypass grafting and valve or transplant procedures, represent a further population of interest, given the substantial psychological burden associated with major surgery, prolonged recovery, and, in the case of transplantation, lifelong immunosuppression and uncertainty about graft outcomes [73]. Emerging trial data suggest that treating perioperative depression with escitalopram can improve both mood and quality of life in patients undergoing bypass surgery, although larger studies in surgical and transplant populations are still needed. In heart transplant recipients specifically, depression and anxiety are common in the peri-transplant period and have been associated with reduced adherence to immunosuppressive regimens, a factor of direct relevance to allograft survival, which further reinforces the case for systematic psychological support integrated into transplant follow-up programs [91].

## 10. Future Directions

Several priorities emerge from this body of evidence. First, there is a need for better biomarkers capable of identifying which depressed cardiac patients are at highest risk of adverse cardiovascular outcomes and which are most likely to benefit from targeted antidepressant treatment; inflammatory markers, heart rate variability indices, and platelet reactivity assays are candidates currently under investigation, though none has yet achieved routine clinical utility [99,101,102]. Second, larger and longer-duration trials, ideally powered specifically for hard cardiovascular endpoints rather than for depressive symptom improvement alone, are needed to clarify whether the encouraging signal observed with escitalopram after acute coronary syndrome can be generalized and replicated elsewhere. Finally, the integration of mental health screening and collaborative care pathways into routine cardiology practice, supported by adequately resourced consultation psychiatry services, represents a pragmatic and genuinely achievable step that could meaningfully improve outcomes even in advance of more definitive trial evidence.

## 11. Conclusions

**Figure 1:**
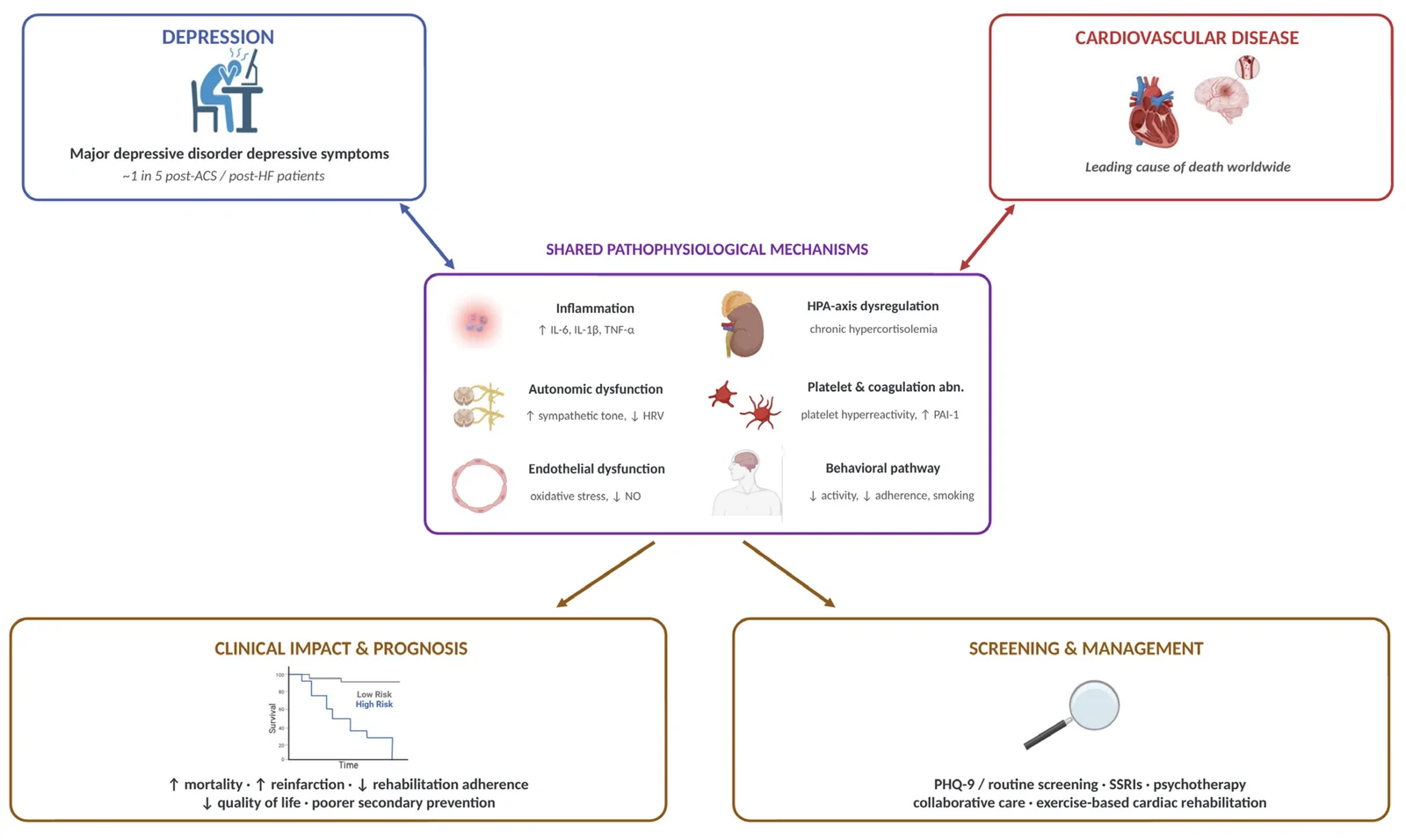
Central Illustration. The bidirectional relationship between depression and cardiovascular disease: shared mechanisms, clinical consequences, and management strategies.

Depression and cardiovascular disease are deeply interconnected by a genuinely bidirectional relationship, sustained by overlapping neuroendocrine, inflammatory, hemostatic, and behavioral pathways, and reinforced by the psychological burden of living with a chronic and potentially life-threatening cardiac condition (Figure 1). Comorbid depression consistently predicts worse cardiovascular outcomes, and yet the path from mechanistic understanding to effective, outcome-modifying treatment remains incompletely mapped, with SSRIs, cognitive behavioral therapy, and exercise-based rehabilitation offering the most consistent evidence of benefit for mood, and more selective evidence for hard cardiac endpoints. Depression should be actively sought, taken seriously as a prognostic factor, and managed with the same rigor applied to other modifiable cardiovascular risks, rather than dismissed as an understandable but clinically secondary reaction to illness. Continued research into risk stratification, novel biomarkers, and population-specific treatment strategies will be essential to properly treat these patients.

## Data Availability

Not applicable. No new data were created or analyzed in this study. Data sharing is not applicable to this article, as this is a narrative review.

## Abbreviations

The following abbreviations are used in this manuscript:

CVD	Cardiovascular Disease
CAD	Coronary Artery Disease
MI	Myocardial Infarction
ACS	Acute Coronary Syndrome
HF	Heart Failure
MDD	Major Depressive Disorder
ANS	Autonomic Nervous System
HPA axis	Hypothalamic–Pituitary–Adrenal Axis
SSRI	Selective Serotonin Reuptake Inhibitor
CBT	Cognitive Behavioral Therapy
PHQ-9	Patient Health Questionnaire-9
